# Real-time Magnetic Resonance-guided Liver Stereotactic Body Radiation Therapy: An Institutional Report Using a Magnetic Resonance-Linac System

**DOI:** 10.7759/cureus.5774

**Published:** 2019-09-26

**Authors:** Aharon M Feldman, Ankit Modh, Carri Glide-Hurst, Indrin J Chetty, Benjamin Movsas

**Affiliations:** 1 Radiation Oncology, Henry Ford Health System, Detroit, USA

**Keywords:** metastatic liver tumors, stereotactic body radiation therapy (sbrt), mri guidance, hepatocellular carcinomas (hcc)

## Abstract

Background

Stereotactic body radiation therapy (SBRT) is a proven and effective modality for treatment of hepatic primary and metastatic tumors. However, these lesions are challenging for planning and treatment execution due to natural anatomic changes associated with respiration. Magnetic resonance imaging (MRI) offers superior soft tissue contrast resolution and the ability for real-time image-guided treatment delivery and lesion tracking.

Objective

To evaluate the plan quality, treatment delivery, and tumor response of a set of liver SBRT cancer treatments delivered with magnetic resonance (MR)-guided radiotherapy on a MR-linear accelerator (MR-linac).

Methods

Treatment data from 29 consecutive patients treated with SBRT were reviewed. All treatments were performed using a step and shoot technique to one or more liver lesions on an MR-linac platform. Patients received 45 to 50 Gy prescribed to at least 95% of the planning target volume (PTV) in five fractions except for two patients who received 27-30 Gy in three fractions. Computed tomography and MRI simulation were performed in the supine position prior to treatment in the free-breathing, end exhalation, and end inhalation breath-hold positions to determine patient tolerability and potential dosimetric advantages of each technique. Immobilization consisted of using anterior and posterior torso MRI receive coils embedded in a medium-sized vacuum cushion. Gating was performed using sagittal cine images acquired at 4 frames/second. Gating boundaries were defined in the three major axes to be 0.3 to 0.5 cm. An overlapping region of interest, defined as the percentage volume allowed outside the boundary for beam-on to occur, was set between 1 and 10%. The contoured target was assigned a 5-mm PTV expansion. Organs at risk constraints adopted by the American Association of Physicists in Medicine Task Group 101 were used during optimization.

Results

Twenty-nine patients, with a total of 34 lesions, successfully completed the prescribed treatment with minimal treatment breaks or delays. Twenty-one patients were treated at end-exhale, and six were treated at end-inhale. Two patients were treated using a free-breathing technique due to poor compliance with breath-hold instructions. The reported mean liver dose was 5.56 Gy (1.39 - 10.43; STD 2.85) and the reported mean liver volume receiving the prescribed threshold dose was 103.1 cm^3^ (2.9 - 236.6; STD 75.2). Follow-up imaging at one to 12 months post treatment confirmed either stable or decreased size of treated lesions in all but one patient. Toxicities were mild and included nausea/vomiting, abdominal pain and one case of bloody diarrhea. Four patients died due to complications from liver cirrhosis unrelated to radiation effect.

Conclusion

SBRT treatment using a gated technique on an MR-linac has been successfully demonstrated. Potential benefits of this modality include decreased liver dose leading to decreased toxicities. Further studies to identify the benefits and risks associated with MR-guided SBRT are necessary.

## Introduction

Hepatocellular carcinoma (HCC) is the fifth most common cancer and the third leading cause of cancer-related deaths worldwide, with a majority of cases in the Asian-Pacific region [1,2]. In the United States, the incidence of HCC has nearly tripled since the 1980s, mostly due to a significant patient population with hepatitis C [3,4]. HCC is now the fastest rising cause of cancer-related deaths [4]. The average age at diagnosis has continued to decline, and most patients with HCC are now diagnosed between the ages of 45 and 60 years [5].

The management of HCC requires a multidisciplinary approach, and the participation of surgical, medical, and radiation oncology is critical. Surgical resection remains a curative option and has consistently demonstrated durable positive outcomes in appropriately selected patients [6]. For non-surgical candidates due to cirrhosis, portal vein thrombosis, or poor Child-Pugh scores, orthotopic liver transplant is an effective option with results similar to resection [5, 6]. For those unable to undergo resection or transplantation, locoregional therapies such as ethanol, radiofrequency, or microwave ablation are recommended. Radiofrequency ablation may offer outcomes similar to resection for patients with small (<2-5 cm) tumors [3,7]. Transarterial embolization and transarterial chemoembolization are excellent options for unresectable HCC, may be used as bridging or downstaging therapies, and have the potential to offer robust overall survival benefits [8]. Transarterial radioembolization with Yttrium-90 can be used in the setting of portal vein thrombosis as it does not cause tissue ischemia. The advent of direct-acting antiviral agent-based therapies has decreased the incidence of hepatitis C virus and by extension of HCC [1,3].

Although traditionally viewed as a palliative treatment, radiation therapy has emerged with improved treatment planning and delivery techniques as a well-tolerated and efficacious therapy option, especially for disease unresponsive to other locoregional therapies. Stereotactic body radiation therapy (SBRT) uses high doses of radiation capable of tumor ablation delivered to precise targets, and its role in the management of hepatic malignancies and liver metastasis offers excellent results [9-12].

Combining liver SBRT with real-time magnetic resonance (MR) guidance has been demonstrated to be an accurate and reproducible treatment modality for these tumors, allowing for superior soft tissue visualization and tumor tracking [9,11-14]. The MRIdian Linac system (ViewRay, Oakwood Village, OH) offers the ability for real-time target tracking and adaptive treatment using MR imaging (MRI) and is the first MR linear accelerator (MR-Linac) system to receive pre-market safety and efficacy clearance from the U.S. Food and Drug Administration [15]. This system contains a double-donut superconducting wide bore magnet with 0.345-T field strength and a 6 -MV flattening filter-free linear accelerator. A double-stacked, double-focused 138-leaf multi-leaf collimator is used for treatment delivery. Combined with a linear accelerator, conformal intensity-modulated radiation therapy plans can be generated while using real-time image guidance to ensure treatment delivery only when the region of interest is in target position. This is important when considering stereotactic treatment to tumors that may change position during respiration, such as those involving the liver [16,17]. Treatment with the MR-Linac system potentially allows for increased target accuracy, tighter margins, increased dose per fraction, and superior dose constraints. We report our initial clinical experience treating hepatic tumors with MR-guided gated radiation therapy on an MR-Linac platform.

## Materials and methods

Patient population

We retrospectively reviewed 29 patients treated on the MRIdian linear accelerator with a step and shoot technique at our institution between August 2017 and October 2018. All patients had one or more biopsy-proven primary or metastatic unresectable liver lesions. Informed written consent was obtained prior to treatment. Treatment volume data, dosimetric values, simulation techniques, toxicity scores, and follow-up information from completion notes, clinic visits, and repeat imaging were reviewed after obtaining institutional review board approval.

Simulation

**Figure 1:**
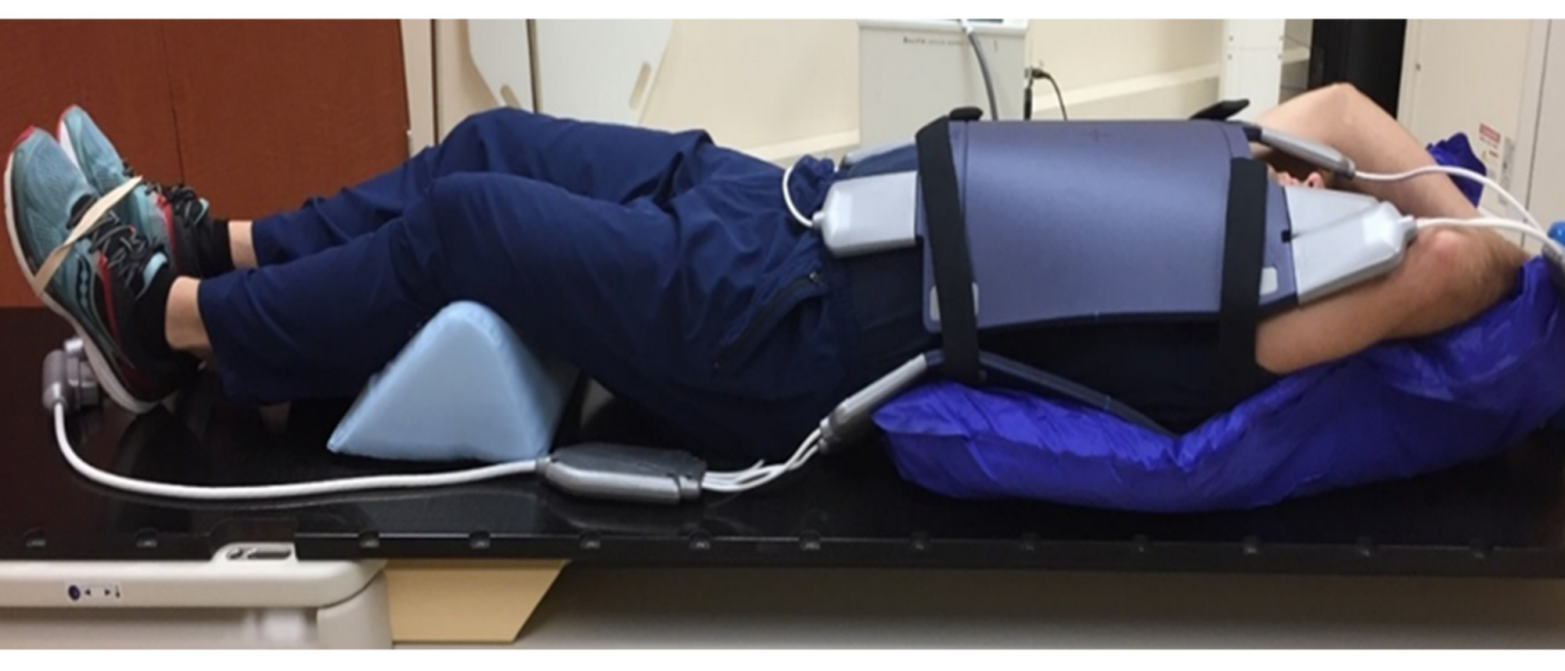
Schematic of patient setup for MR simulation. Immobilization devices consist of anterior and posterior torso MRI receive coils embedded in a medium-sized vacuum cushion.

Computed tomography (CT) and MRI simulation were performed in the supine position prior to treatment in the free breathing, end exhalation, and end inhalation breath-hold positions to determine patient tolerability and potential dosimetric advantages of each technique. CT simulation was performed with intravenous contrast and 3 mm slices on a large bore, 16-slice CT scanner, and an appropriate isocenter was placed with physician guidance. This was immediately followed by the MR simulation performed on the MR-Linac unit. Care was taken to ensure proper MR-Linac bore and patient clearance. Immobilization was achieved using a medium-sized Vac-Q-Fix Cushion (Qfix, Avondale, PA) with anterior and posterior torso MRI receiver coils, knees flexed and supported on a blue angle sponge, feet banded together, and arms raised above the head with hands holding a foam ring (Figure 1) [18].

Volume delineation

Normal structures and tumor volumes were contoured on the pretreatment CT scan obtained at the time of simulation and fused with the MR simulation and other diagnostic images at the physician’s discretion. Volumes were contoured on Eclipse (Varian, Palo Alto, CA) software and subsequently transferred to the ViewRay treatment planning system. Normal structures included the liver, heart, stomach, great vessels, lungs, kidneys, and small bowel, if applicable. The gross tumor volume was defined as the region of discernable disease identified on diagnostic imaging. A clinical target volume was added at the discretion of the physician to cover additional regions of potential microscopic disease, and an additional 5-mm margin was added to the gross tumor volume or clinical target volume, when applicable, to create the planning target volume (PTV).

Treatment planning

Treatment planning was performed using ViewRay software. CT and MRI scans from simulation were imported for planning. A Monte Carlo treatment planning algorithm to a single isocenter was used to plan treatment to the PTV. Between six and 16 beams were used for treatment. Doses ranged from 45 to 50 Gy in five fractions, except for three patients who were planned for three fractions. Dose was prescribed to at least 95% of the PTV. American Association of Physicists in Medicine Task Group 101 (AAPM TG-101) treatment planning criteria for organs at risk were used for treatment planning purposes [19].

Treatment delivery

**Figure 2:**
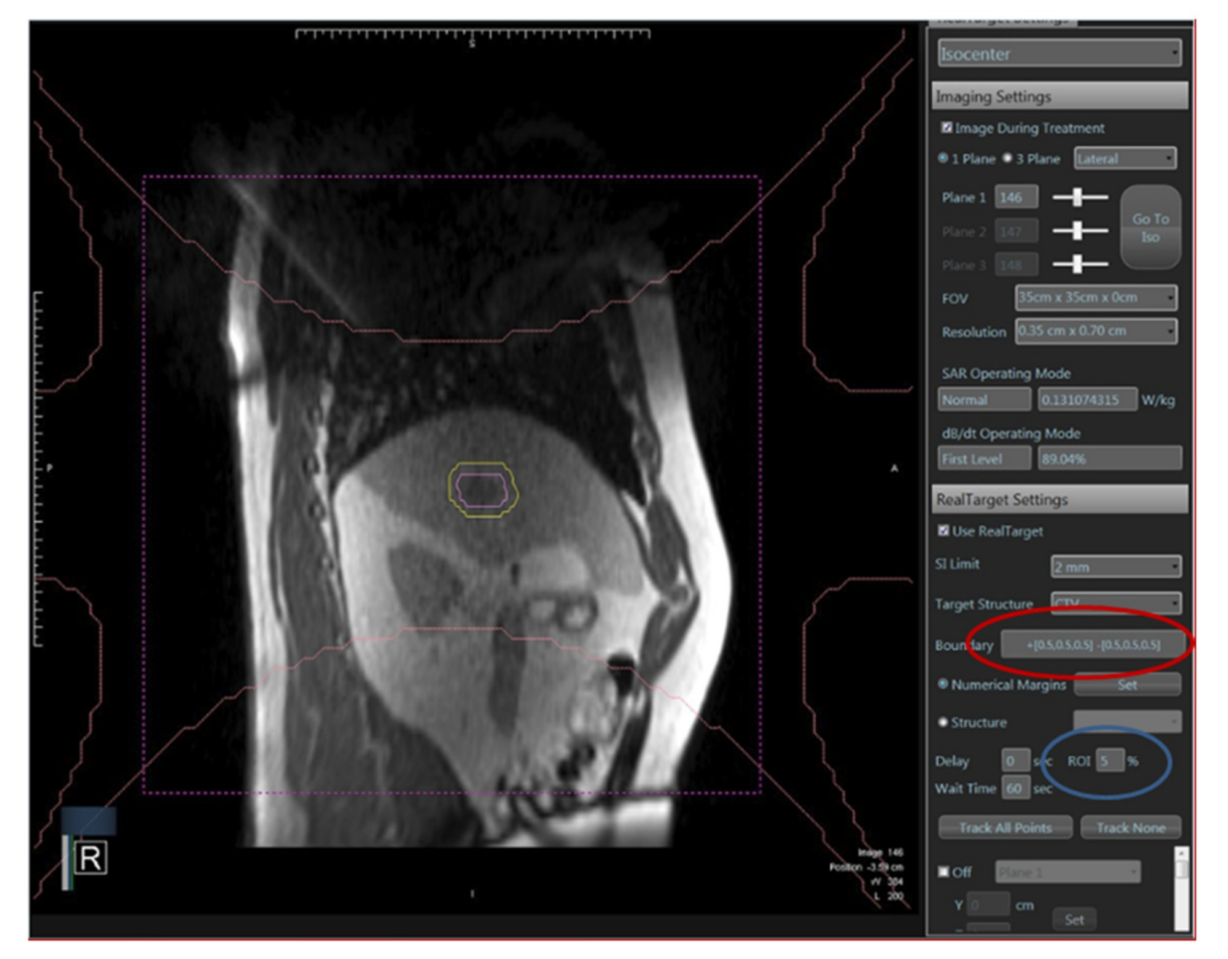
Boundary expansion (circled in red) defining the size of gating window. Also shown is the %ROI (circled in blue): percentage of the target volume that could be outside of the boundary without triggering a beam hold, typically ranging from 1 to 5%.

Sixteen tumors were treated to a total dose of 45 Gy in five fractions, 15 tumors received 50 Gy in five fractions, and the remaining three were treated in three fractions to doses ranging from 27 to 42 Gy. Real-time image guidance via a sagittal cine acquisition at 4 frames/second was implemented by assigning a boundary that would deactivate the treatment beam if violated. Boundaries were assigned to the vertical, horizontal, and axial planes and ranged between 0.3 and 0.5 mm. A percentage of the target region of interest, defined as a percentage of the target volume that could be outside of the boundary without triggering a beam hold, ranged from 1% to 5% (Figure 2).

Based on observed target motion and patient tolerances, the reference breathing phase (end-inhale, end-exhale, or free breathing) was selected and treatment was delivered using a gating technique.

Follow-up

Patients were scheduled for a clinic visit and follow-up imaging 2-3 months after completing treatment. Follow-up status was assessed based on patient evaluation dictated at return visits and imaging performed at that time.

## Results

The 29 patients (19 males and 10 females) with unresectable liver tumors (total of 34 lesions) underwent SBRT with minimal treatment breaks or delays. The case distribution included 26 patients with HCC, two with cholangiocarcinoma, and one with metastatic colon cancer. Twenty-one patients were treated at end-exhale and six were treated at end-inhale using verbal coaching to improve treatment efficiency. Two patients were treated free-breathing due to poor compliance with the breath-hold technique. One patient was also treated with an adaptive technique. All patients completed their treatment course.

SBRT plans adhered to AAPM TG-101 treatment planning criteria for organs at risk. The mean liver dose was 5.56 Gy (1.39-10.43; standard deviation [SD] 2.85), and the mean liver volume receiving the prescribed threshold dose was 103.1 cm^3^ (2.9-236.6 [SD 75.2]). The mean number of beams during treatment was 10.8 (6-16 [SD 2.4]), and the mean beam segments used was 31.1 (10-64 [SD 12.5]). The mean monitor units (MU) per fraction was 2538.9 MU (1549.1-5737.4 [SD 1103.5]). The average treatment time was 34 minutes.

Follow-up imaging with either CT with contrast and liver protocol or MRI abdomen with contrast ranged from one to 12 months post treatment and confirmed either stable or decreased size of all but one treated lesion. Toxicity information, obtained by reviewing radiation treatment completion notes and follow-up clinic visit notes, included nausea and vomiting in one patient and one case of abdominal pain with bloody diarrhea, which required a brief treatment break and resolved without any intervention. Four patients died due to complications from liver cirrhosis unrelated to radiation effect.

## Discussion

While use of SBRT in clinical practice has more than doubled over the past decade [20,21], its use for tumors of the liver has yet to match that in the lung primarily because of technical challenges [17,22]. Most significantly, movement of the liver due to the physiologic breathing cycle and poor visibility with contemporary on-board imaging make these targeted treatments challenging [16,17,22,23]. The MR-Linear model used in this report of our institutional experience treating primary and secondary liver cancers addressed these technical challenges by offering superior imaging and allowing for real-time tumor tracking. Changes in intrinsic organ anatomy or intrafraction excursions were also negated with this modality.

**Figure 3:**
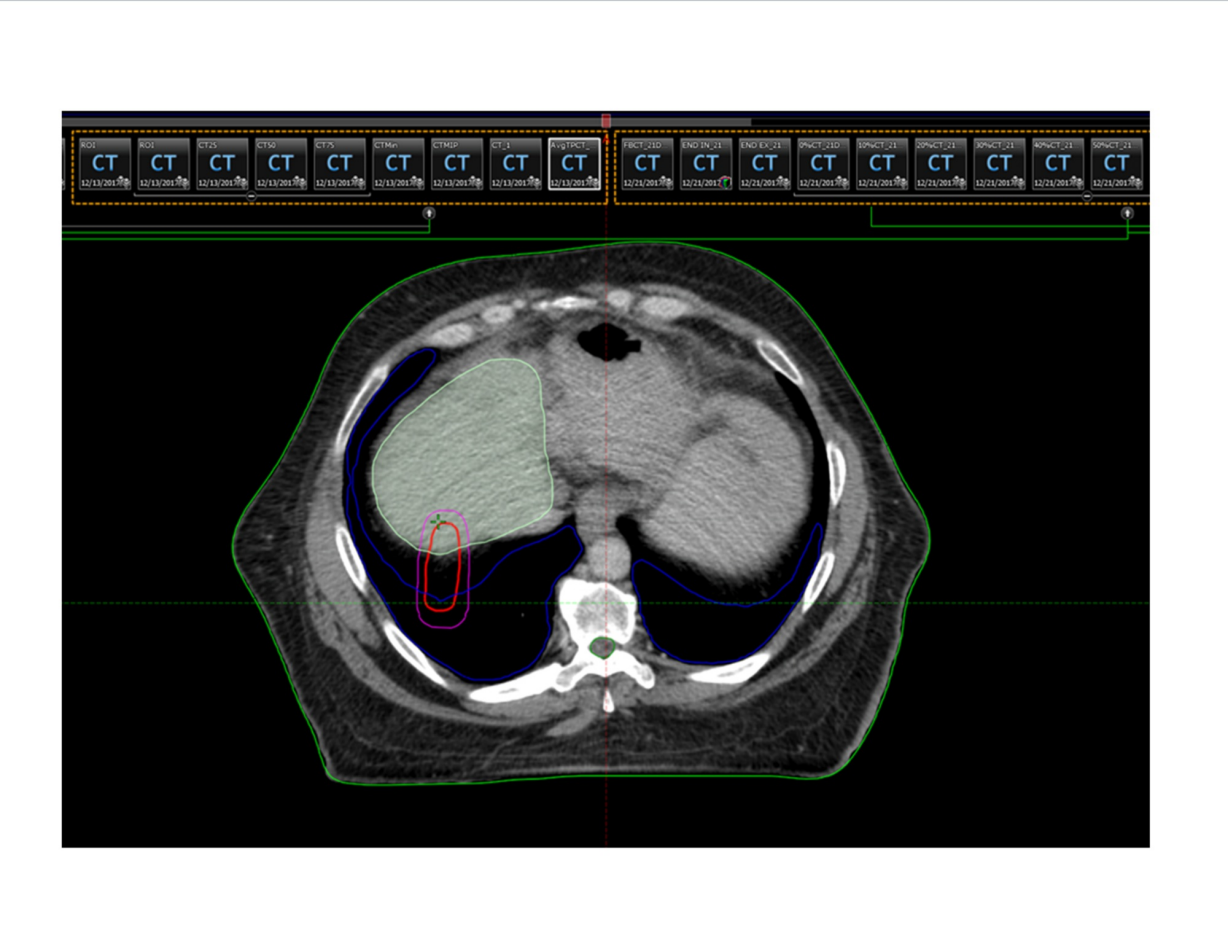
Volume encompasses all phases of the respiratory cycle in the creation of an internal target volume (ITV), necessitating a larger contour (outlined in red).

**Figure 4:**
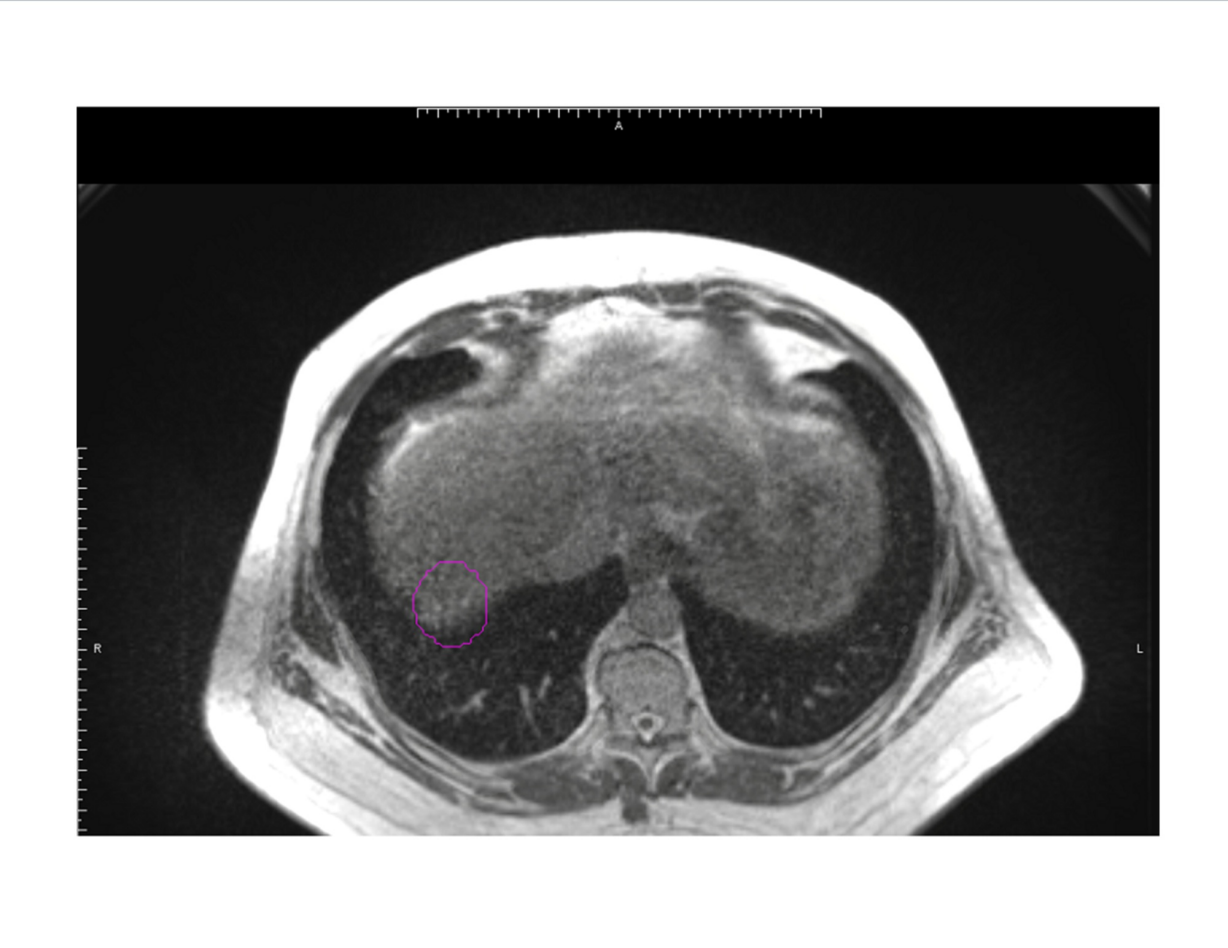
Due to tumor tracking and superior soft tissue resolution, the planned volumes for the MR-Linac are significantly tighter (outlined in magenta).

The ability to track the tumor in real time allows for accurate volume delineations without the need for generous margins, potentially allowing for higher dose per fraction regimens [14]. This is especially important as tumors treated to higher doses have exhibited superior local control outcomes, with >132 Gy biological equivalent dose showing the greatest benefit [11]. The following figures illustrate a segment 5 liver lesion contoured for planning on a traditional linac (Figure 3) and on the MR-Linac (Figure 4).

As demonstrated, the contoured volume on Figure 3 encompasses all phases of the respiratory cycle in the creation of an internal target volume, necessitating a larger contour with a volume of 10.7 cm^3^. With the added 5 mm PTV expansion, the volume measures 40.8 cm^3^. Due to tumor tracking and superior soft tissue resolution, the planned volumes for the MR-Linac on Figure 4 are significantly tighter, with a gross tumor volume of 7.17 cm^3^ and a PTV of 25.14 cm^3^. The smaller treatment volumes in our cohort contributed to the gradual increase from 9 Gy to 10 Gy per fraction as treatment experience continued to develop.

An additional benefit of enhanced soft tissue contrast is the allowance for fiducial-free lesion targeting at an MRI frame rate of 4 frames/second, thus avoiding the potential complications of an invasive procedure. On-board MRI is also now being used to facilitate adaptive planning [24]. In our cohort, one patient was successfully treated with an adaptive technique.

Although we did not directly compare treatment plans generated for delivery on a traditional linear accelerator with our patient cohort, our plans consistently met target mean liver doses similar to those expected on a traditional linear accelerator, a benefit that proved challenging for the MR-guided ^60^Co system, largely due to differences in beam penumbra and multi-leaf collimator design [14]. Additionally, due to the MR-Linac system’s capability of delivering dose at 600 MU/min, treatment time would potentially be significantly shorter than those on a traditional linac. The average treatment time of our patient cohort was 34 minutes, including patient setup, on-board imaging, setup adjustments, delivery, and exit. This timing is comparable to previous cohorts treated on a linac. Shorter treatments can significantly impact the applicability of this modality, as a longer duration of treatment would understandably exclude potential treatment candidates.

MR-Linac based treatment presents several challenges [14,15,25,26]. The current system is limited to 6X-flattening filter-free photons, which can restrict the treatment of deep-seated and larger lesions that require higher beam energies for deeper penetration to yield clinically acceptable plans. Furthermore, the magnetic field generated by the MRI unit can potentially affect the function of the magnetron and port circulator [15]. It can also alter the direction of electrons generated from the photon beam, increasing dose heterogeneity. Electrical fields created by the split gradient coils of the MR system must be considered when creating treatment plans as well [27]. High radiofrequency energy generated from the linac can influence MR image quality [15]. The 0.35-T magnet, although weaker than traditional 1.5-T or 3-T magnets, reduces some of these concerns, albeit at the expense of image quality. Nevertheless, gated MR-Linac delivery was well tolerated and all patients completed their SBRT course, with five patients undergoing two separate targeted treatments.

Robust MRI safety protocols must be observed throughout treatment planning and delivery [28]. Burns are a common injury reported in the MRI environment and potential patients must be thoroughly screened before treatment for embedded implants, devices, or other metallic objects [29,30]. Vulnerable anatomy can be potentially damaged due to vibrations from the MR unit. Hearing protection is required for all patients.

Limitations to this report include the relatively short follow-up time, a small patient population from a single institution, and the retrospective nature of the review. As our experience continues to grow and patient follow-up data mature, a more robust experience can be reported and results can be confirmed.

## Conclusions

SBRT treatment to the liver using an MR-Linac appears promising. Its use allows for potentially tighter margins and reduced toxicity, possibly leading to improved patient outcomes. Further follow-up is needed to identify potential toxicities associated with treatment and more studies are warranted to identify the benefits and risks associated with MR-guided SBRT.
